# Routine binocular examination of young Taiwanese adults as a predictor of visual behavior performance

**DOI:** 10.1186/s12886-022-02731-1

**Published:** 2023-02-01

**Authors:** Shyan-Tarng Chen, Kuo-Chen Su, Po-Hsin Wang, Xiang-Yin Zhong, Ching-Ying Cheng

**Affiliations:** 1 Department of Optometry, Chung Shan Medical University, Taichung, 402 Taiwan; 2 Department of Ophthalmology, Chung Shan Medical University Hospital, Taichung, 402 Taiwan

**Keywords:** Binocular visual function, Binocular accommodative examination, Visual behavior performance

## Abstract

**Background:**

Morgan and Scheiman’s Optometric Extension Program (OEP) expected binocular vision findings have longstanding use in optometry. With technological advances, the demands and standards of binocular function have changed. This study aimed to investigate which binocular visual functions can effectively predict visual behavior performance.

**Methods:**

Participants aged 15–24 years were recruited from two colleges and two universities. After completing the CSMU-Visual Behavioral Performance questionnaire (CSMU-VBP, with four components: near work, visual perception, visual comfort, and whole-body balance), participants were divided into symptomatic and asymptomatic groups based on questionnaire findings (cutoff: < 12 vs. ≥ 12 symptoms). Then a 24-step binocular visual examination was undertaken. Data were analyzed with one-sample, Student’s, and paired t-tests. Additionally, receiver operating characteristic analysis was used to determine the predictors of binocular visual function required for near work, visual perception, visual comfort, and body balance dimensions.

**Results:**

Among 308 participants, 43 (14%) and 265 (86%) were symptomatic and asymptomatic, respectively. Among the 46 participants with abnormal binocular vision, 36 (78%) reported that they had no obvious symptoms. The commonest dysfunctions were accommodative excess and convergence excess. Most of the binocular visual findings significantly diverged from traditional normal values: amplitude of accommodation, as well as base-in prism to break and recovery points at distance were higher than traditional normal values, whereas others were lower than traditional normal values. Total CSMU-VBP scores indicated that the asymptomatic and symptomatic groups had significant differences in DBO recovery (t = 2.334, *p* = 0.020) and BAF (t = 1.984, *p* = 0.048). Receiver operating characteristic curve analysis yielded the following binocular visual functional cutoff points: near work (DBO blur < 7, DBO recovery < 5.5), visual perception (MAF < 10.5, BAF < 10.25), visual comfort (DLP <  − 2.25, DBI break > 11.5, NBI blur > 15, NBI break > 17.5, NBI recovery > 13, NPC < 5.75), and body balance (NFD_H >  − 0.5, gradient AC/A [minus] > 2.25, NPC < 4.75).

**Conclusions:**

The mean values of binocular visual function among young Taiwanese adults were statistically different from traditional normative values. Further research is required to confirm whether these findings reflect impaired binocular vision or stringent criteria. Assessments of binocular visual function, especially binocular accommodation sensitivity, are crucial in routine optometric examination.

**Supplementary Information:**

The online version contains supplementary material available at 10.1186/s12886-022-02731-1.

## Introduction

In the context of normal eye structure, the ability to combine evaluations of binocular vision, large visual field overlap, and retinal cell potential with comprehensive brain analysis has optimized imaging resolution, facilitating the ability to perceive three-dimensional visuospatial and stereoptic information for further assessing (and potentially enhancing) fine motor coordination and manipulation abilities [1–3]. Binocular visual dysfunction can be associated with many symptoms, such as blurred vision, headache, eye strain or discomfort, intermittent diplopia, inattention, eye rubbing, excessive blinking, and photophobia [4–9]. Such dysfunction and symptomatology can lead to abnormal visual behavior performance, such as reading with finger assistance, skipping or missing, letter reversal, or lack of interest in near vision. Moreover, binocular visual problems may lead to other related physiological or perceptual problems, including issues with peripheral perception [10], athletic ability [11], visuospatial discernment, and sense of direction [12]. In the study reported herein, we defined visual behavior performance as near work, visual perception, visual comfort, and body balance.

The standards and expected values of Morgan and Scheiman’s Optometric Extension Program (OEP) [1, 13], which are frequently cited in clinical binocular vision examinations in optometry, have been well established since 1944. Over time, with scientific and technological advancements, habits and functional demands associated with vision have changed. Additionally, in light of genetic and regional variations, the suitability of these standards values requires re-evaluation [14–20]. Age is an important factor that affects binocular vision, including the elements of contrast sensitivity [21], visual acuity [22], accommodation [23], and vergence [24–26]. Furthermore, aging reduces the amplitude and time of vergence peak velocity [27] and phoria adaptation [28]; such changes are often detected in patients with binocular dysfunction, especially in patients with convergence insufficiency [29–32]. However, the Morgan and Scheiman OEP standards were not established with age-specific considerations.

Besides age, optometrists should pay attention to the correlations between visual function, brain injury [33], dry eye [34–36], migraines [37], sleep disturbance [38, 39], dyslexia [40], inattention [41, 42], work in high-tech industries [43], and athletic activity [44]. Moreover, with the emergence of virtual reality technology in recent years, associated problems with motion sickness are yet unsolved; this has been shown to be associated with binocular vision problems, but to our knowledge, no published studies have investigated this problem in Taiwan among Taiwanese populations [14]. Therefore, the application of traditional standards for related clinical diagnoses in Taiwan may be problematic. This study investigated binocular visual function among young adults in Taiwan and compared the findings with traditional standard values. Furthermore, the study aimed to further analyze binocular visual function tests to determine accurate standard requirements for different visual tasks.

## Materials and methods

This cross-sectional study was conducted from November 18, 2019, to May 30, 2020. The study protocol was reviewed and approved by the institutional review board of Chung Shan Medical University Hospital (approval no. CS19110). Additionally, the study strictly adhered to the principles of research ethics specified in the Declaration of Helsinki and its later amendments, and this article followed the STROBE guidelines [45].

### Participants

Non-probability and convenience sampling techniques were used, and study participants were recruited from two colleges and two universities. According to information from the Taiwan Ministry of Education, 98.94% of young people in Taiwan are studying in colleges and universities, that is, most Taiwanese youths and young adults, aged 15–24, are college and university students. A total of 327 young individuals, aged 15 to 24 years, consented to participate. The exclusion criteria were as follows: refractive errors of sphere ≤  − 6.00 D or >  + 1.00 D; astigmatism ≤  − 1.00 D; long-term use of contact lenses; previous eye or brain surgery; and ophthalmic, metabolic, immune, physiological, or psychological diseases. Additionally, patients with severe visual complaints which had great influence on their quality of life were also excluded. All participants were confirmed to be healthy through objective and subjective screening procedures. Among those who consented to participate, 10 had incomplete questionnaires and eye examinations, five had best corrected visual acuity ratios ≤ 1.0, one had Ménière's disease, one had Tourette's disease, one had amblyopia, and one had strabismus, and the final analysis included 308 participants. The Kolmogorov–Smirnov test revealed that the participants’ spherical equivalent power data were under normally distributed (right eye: D = 0.068, *p* = 0.148; left eye: D = 0.062, *p* = 0.236). The participants’ characteristics are summarized in Table 1.

**Table 1:** Basic information of the participants

	**Total**	**symptomatic**	**asymptomatic**	**t**	**p**
Subjects	308	43	265	–-	–-
Age	18.72 ± 1.672	18.49 ± 1.549	18.75 ± 1.691	0.969	0.333
Gender(M/F)	83/225	7/36	76/189	Χ^2^ = 2.036	0.154
Spherical equivalent (SE: Right eye, OD)	-3.172 ± 2.594	-3.457 ± 2.448	-3.126 ± 2.588	0.775	0.439
Spherical equivalent (SE: Left eye, OS)	-3.128 ± 2.627	-3.535 ± 2.349	-3.062 ± 2.639	1.095	0.274
SE OD v.s.OS	t = -0.935, *p* = 0.351	t = 0.746, *p* = 0.460	t = -1.406, *p* = 0.161	–-	–-
Refractive errors group	Emmetropic:82 (26.6%) Myopia: 226 (73.4%) Astigmatic: 140(45.5%)	Emmetropic:11 (25.6%) Myopia: 32 (74.4%) Astigmatic: 20(46.5%)	Emmetropic:71 (26.8%) Myopia: 194 (73.2%) Astigmatic: 120(45.3%)	–-	–-
Total CSMU-VBP Score	6.440 ± 5.091	16.256 ± 3.381	4.849 ± 3.174	-21.659	0.001**
Near Work	3.399 ± 3.154	9.326 ± 2.485	2.437 ± 1.984	-20.337	0.001**
Perception	2.104 ± 2.211	5.744 ± 2.441	1.513 ± 1.493	-11.038	0.001**
Comfort	3.036 ± 2.391	6.512 ± 2.492	2.472 ± 1.836	-10.193	0.001**
Body Balance	1.159 ± 1.323	2.837 ± 1.632	0.887 ± 1.038	-7.592	0.001**

^*^p < 0.05, **p < 0.01

### Research materials

The assessment procedures used in this study were non-invasive, non–risk-conferring, and involved no drug administration. Accordingly, the research tools and examination items were divided into two parts:

#### Binocular visual function examination

To ensure accurate measurement results, possible confounding factors, such as laboratory brightness, visual target distance, and subjective differences in measurement tools and equipment operators, were controlled. Additionally, to avoid any potential sources of bias, each test was performed by the same optometrist. The binocular visual function examination and materials included:

- Refraction: Shin-NiPon Openfield Refraction (Tokyo, Japan)
- Subjective refraction: Topcon Manual Phoropter VT-10(Tokyo, Japan)
- Distance visual acuity and distance vergence range: Digital Chart System VM-VLC-1900
- Near visual acuity and near vergence range, negative relative accommodation (NRA), and positive relative ac-commodation (PRA): TMVC Near Point Test Card, plus lens power is added binocularly, 0.25D at a time.
- Horizontal and vertical phoria: Von Graefe, Risley rotating prisms on phoropter.
- Accommodation amplitude (AA): Donder’s push-up method with the RAF near point ruler
- Monocular and binocular accommodation facility (MAF, BAF), convergence facility, near-fixation disparity (NFD): ± 2.00 D flippers for accommodation, 8BI/8BO flippers for vergence, and the Saladin near point balance card
- near-point convergence (NPC), near-point accommodation (NPA): Royal Air Force(RAF) ruler with a vertical line target.
- Fusional vergence range: Risley rotating prisms on phoropter; Fusional vergence facility: 12 PD base-out and 3 PD base-in flipper performed at 40 cm.

#### Visual behavior performance questionnaire

Notably, most questionnaires used to investigate or query binocular function focus on near work or visual symptoms, and few such questionnaires have focused on visual behavior indicators, such as visual perception, walking posture, or balance. The CSMU-Visual Behavioral Performance questionnaire (CSMU-VBP) was developed by this study’s investigators and was based on the convergence insufficiency symptom survey(CISS) [46], college of optometrists in vision development quality of life checklist (COVD-QoL) [47], and the students' visual status questionnaires [48]. The content of CSMU-VBP(48 questions) was screened and analyzed by Analytical Hierarchy Process(AHP) by three optometric experts, and has been widely used in binocular vision and visual training research in Taiwan [49–52]. Factor analysis divided the questionnaire (overall Cronbach's alpha = 0.851) into four dimensions (each question may be calculated repeatedly), with 25 questions pertaining to near-vision work (Cronbach's alpha = 0.837), 20 on perception (Cronbach's alpha = 0.780), 14 on comfort (Cronbach's alpha = 0.705), and 12 on postural balance (Cronbach's alpha = 0.775) [49].

According to CSMU-VBP responses, participants were divided into the asymptomatic (good visual performance, total score < 12) and symptomatic (poor visual performance, total score ≥ 12) groups by using the lowest quartile as the cutoff criterion [53]. Logistic regression analysis indicated that the accuracy of all binocular visual function examinations in predicting symptomatic or asymptomatic partcipatns (according to questionnaire responses) was as high as 86.4%, especially with regard to NLP (exp(β) = 1.102, *p* = 0.040), DBI recovery (exp(β) = 1.287, *p* = 0.025), BAF (exp(β) = 0.853, *p* = 0.032), and AA (exp(β) = 1.129, *p* = 0.025) [49].

### Data and statistical analysis

One-sample, Student’s, and paired t-tests, the Kolmogorov–Smirnov test, logistic regression analysis, and receiver operating characteristic (ROC) curve analysis were performed, and data were analyzed using SPSS Statistics for Windows, version 26.0 (Armonk, NY, USA); a *p-*value < 0.05 was considered statistically significant.

## Results

Of 308 participants, 83 were males, and 225 were females. The mean age was 18.7 ± 1.7 years. There was no significant difference between the gender in terms of spherical equivalent power (male: − 3.47 ± 2.51 D, female: − 3.25 ± 2.59 D; t = 0.766, *p* = 0.444); therefore, differences between the gender were not analyzed further.

Among the 308 participants, 43 (14%) were classified as symptomatic, and the remaining 265 (86%) were asymptomatic. There was no significant difference between the symptomatic and asymptomatic groups in terms spherical equivalent power according to eye laterality (right eye: t = 0.775, *p* = 0.439; left eye: t = 1.095, *p* = 0.274). There was also no significant difference between the left and right eyes in terms of spherical equivalent power according to stud group (total: t =  − 0.935, *p* = 0.351; symptomatic: t = 0.746, *p* = 0.460; asymptomatic: t =  − 1.406, *p* = 0.161). The total CSMU-VBP score (t =  − 21.659, *p* = 0.001), near work (t =  − 20.337, *p* = 0.001), perception (t =  − 11.038, *p* = 0.001), comfort (t =  − 10.193, *p* = 0.001), and body balance (t =  − 7.592, *p* = 0.001) dimensions yielded significant differences between the symptomatic and asymptomatic groups (Table 1).

### Binocular visual function and visual behavior performance in young Taiwanese adults

**Fig. 1:**
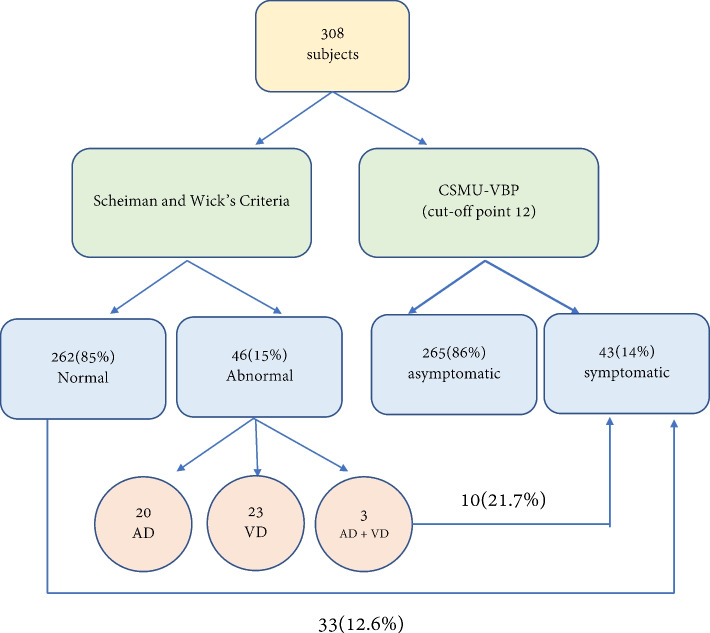
Frequency of Scheiman and Wick’s criteria and CSMU-VBP

According to Scheiman and Wick’s criteria [1], the proportion of normal binocular vision in this study was ascertained to be 85% (*n* = 262), and the frequency of abnormal accommodation and convergence was 15% (*n* = 46). Among all the participants, 20 only had accommodation dysfunction (AD, 6.5%), among whom excessive accommodation was the commonest problem (*n* = 13, 4.2%). Twenty-three participants only had vergence dysfunction (VD, 7.5%), among whom excessive convergence was the commonest problem (*n* = 12, 3.9%). Three participants simultaneously had AD and VD (1%; Table 2 and Fig. 1).

**Table 2:** Frequency of accommodation and vergence anomalies according to Scheiman and Wick’s criteria [1]

	Frequency	Percentage	Asymptomatic		Symptomatic	
Normal	262	85%	229	87.4%	33	12.6%
Accommodative dysfunction (AD)	20	6.5%	15	75%	5	25%
Accommodative excess (AE) **F:** MAF < 6 cpm with + 2.00D lenses C: BAF < 3 cpm, NRA < 1.50D	13	4.2%	11	84.6%	2	15.4%
Accommodative insufficiency (AI) **F:** MAF < 6 cpm with ± 2.00D lenses **C:** BAF < 3 cpm, PRA < 1.25D, NRA < 1.50D	4	1.3%	3	75%	1	25%
Accommodative infacility **F:** Reduced AA: 2.00D < Minimum AA (15–0.25 × age) **C:** MAF < 6 cpm, BAF < 3 cpm, PRA < 1.25D	2	0.6%	0	0%	2	100%
Accommodative excess (AE) and accommodative infacility	1	0.3%	1	100%	0	0%
Vergence dysfunction(VD)	23	7.5%	18	78%	5	22%
Convergence excess(CE) **F:** Significative exophoria at near vision (≥ 6Δ),greater than far vision **C:** PFV at near ≤ 11/ 14/ 3Δ(at least one item meets); NPC ≥ 6 cm, VF ≤ 13 cpm, BAF < 3 cpm, NRA < 1.50D	12	3.9%	9	75%	3	25%
Convergence insufficiency(CI) **F:** Significant esophoria at near vision (≥ 1Δ), greater than far vision **C:** NFV at near ≤ 8/ 16/ 7Δ (at least one item meets); VF ≤ 13 cpm, BAF < 3 cpm, PRA < 1.25D	4	1.3%	2	50%	2	50%
Basic exophoria **F:** Significant exophoria at far and near vision of equal amount(deviations within 5 Δ of one another are considered equal) **C:** PFV at far ≤ 4 / 10/ 5Δ and ≤ 11/ 14/ 3Δ at near (At. least one item meets); NPC ≥ 6 cm, VF ≤ 13 cpm, BAF < 3 cpm,NRA < 1.50D	3	1.0%	3	100%	0	0%
Basic esophoria **F:** Significant esophoria at far and near vision of equal amount(deviations within 5 Δ of one another are considered equal) **C:** NFV at far ≤ X / 3/ 1Δ and ≤ 8/ 16/ 7Δ at near(at. least one item meets); VF ≤ 13 cpm, BAF < 3 cpm, PRA < 1.25D	4	1.3%	4	100%	0	0%
AD + VD	3	1.0%	3	100%	0	0%
Accommodative insufficiency and convergence insufficiency (AI + CI)	1	0.3%	1	100%	0	0%
Accommodative insufficiency (AI) and basic esophoria	1	0.3%	1	100%	0	0%
Accommodative excess (AE) and accommodative infacility and basic esophoria	1	0.3%	1	100%	0	0%
Total			265	86%	43	14%

*F* Fundamental signs, *C* Complementary signs

Among the 46 participants with abnormal binocular visual function, 78% (36 participants) reported that they had no obvious symptoms (Table 2), three had exophoria, four had esophoria, and three had abnormal accommodation and vergence. Half of the participants with impaired accommodative facility or convergence-insufficient cohesion had symptoms. In particular, among the 262 participants with normal binocular visual function that were classified according to traditional diagnostic criteria, 33 (12.6%) had visual complaints, and only 10 (21.7%) of the 46 participants with abnormal visual function complained of poor visual behavior performance, indicating that the patients who reported that they had visual behavior problems may not have had binocular visual problems (Fig. 1). Due to the fact that different types of visual work require different binocular visual functions; the clinical characteristics and Scheiman and Wick's diagnostic criteria for binocular visual dysfunction can be further considered.

### Comparison of binocular visual function values of Morgan and Scheiman’s OEP

The mean values of distance phoria; near phoria; gradient AC/A plus and minus; DBI break and recovery; DBO blur, break, and recovery; NBI blur, break, and recovery; NPC, MAF, BAF, NRA, PRA, and AA, all diverged significantly from the Morgan and Scheiman standards. In particular, the mean values of DBI break, recovery, and AA were all higher than the Morgan and Scheiman standard, and the remainder of the assessed criteria were lower than the standard values.

Compared with the expected OEP values, except for DBO blur, there were statistically significant differences in distance phoria, near phoria, DBI break and recovery, DBO break and recovery, NBI blur, break, and recovery, as well as NRA and PRA. Among the investigated variables with significant differences (except for DBI break, which yielded a higher mean value than the expected OEP value), the mean values of the rest of the items were lower than the expected values (Table 3).

**Table 3:** Comparison between binocular visual function and traditional standard values

Examination	Total (*n* = 308) Mean ± SD	Scheiman’s normal range	*t* *p*-value	95%CI**(lower ~ upper)** comparison	OEP Expected finding	*t* *p*-value	95%CI**(lower ~ upper)** comparison
DLP	1.79 exophoria ± 3.26	1 exophoria ± 2	-4.310 < 0.001	-1.157 ~ -0.433 ↓	0.5	-7.034 < 0.001	-1.657 ~ -0.933 ↓
NLP	5.85 exophoria ± 6.73	3 exophoria ± 3	-7.601 < 0.001	-3.639 ~ -2.143 ↓	6	0.287 0.697	-0.638 ~ 0.857 ≈
NFD_H	0.38 exophoria ± 2.14	-	-	-	-	-	-
Gradient AC/A(plus)	2.57 ± 4.93	4 ± 2	-4.338 < 0.001	-2.000 ~ -1.076. ↓	-	-	-
Gradient AC/A(minus)	2.59 ± 4.67	4 ± 2	-4.127 < 0.001	-2.138 ~ -1.007. ↓	-	-	-
DBI_break	11.21 ± 3.67	7 ± 3	20.123 < 0.001	3.744 ~ 4.592 ↑	9	10.502 < 0.001	1.774 ~ 2.592. ↑
DBI_recovery	4.39 ± 2.55	4 ± 2	2.610 0.0387	0.092 ~ 0.661. ↑	5	-4.306 < 0.001	-0.907 ~ -0.338. ↓
DBO_blur	6.46 ± 7.11	9 ± 4	-6.099 < 0.001	-3.253 ~ -1.666. ↓	7	-1.140 0.186	-1.253 ~ 0.334 ≈
DBO_break	17.22 ± 7.81	19 ± 8	-3.852 0.0143	-2.268 ~ -0.851. ↓	19	-4.388 < 0.001	-2.968 ~ -1.213. ↓
DBO_recovery	6.2 ± 4.04	10 ± 4	-16.003 < 0.001	-4.276 ~ -3.339. ↓	10	-16.003 < 0.001	-4.276 ~ -3.339. ↓
NBI_blur	6.81 ± 7.24	13 ± 4	-15.100 < 0.001	-7.004 ~ -5.389. ↓	14	-17.537 < 0.001	-8.004 ~ -6.389. ↓
NBI_break	18.36 ± 6.37	21 ± 4	-7.287 < 0.001	-3.332 ~ -1.915. ↓	22	-10.064 < 0.001	-4.332 ~ -2.915. ↓
NBI_recovery	11.16 ± 5.49	13 ± 5	-5.743 < 0.001	-2.400 ~ -1.175. ↓	18	-21.806 < 0.001	-7.400 ~ -6.175. ↓
NBO_blur	4.23 ± 7.21	17 ± 5	-30.766 < 0.001	-13.455 ~ -11.838. ↓	15	-25.901 < 0.001	-11.455 ~ -9.838. ↓
NBO_break	15.78 ± 6.85	21 ± 6	-13.005 < 0.001	-5.940 ~ -4.379. ↓	21	-13.005 < 0.001	-5.940 ~ -4.379. ↓
NBO_recovery	7.16 ± 5.47	11 ± 7	-12.169 < 0.001	-4.453 ~ -3.213. ↓	15	-24.866 < 0.001	-8.453 ~ -7.213. ↓
NPC_break	6.16 ± 2.68	5 ± 2.5	2.280 < 0.001	1.010 ~ 0.074 ↓	-	-	-
MAF	9.45 ± 5.03	11 ± 5	-5.377 < 0.001	-2.100 ~ -0.975. ↓	-	-	-
BAF	9.93 ± 4.41	10 ± 5	-0.237 0.9193	-1.233 ~ 1.445 ≈	-	-	-
NRA	1.71 ± 0.61	2.00 ± 0.50	-8.600 < 0.001	-0.324 ~ -0.229. ↓	2.00	-8.600 < 0.001	-0.324 ~ -0.229. ↓
PRA	-1.97 ± 1.29	-2.37 ± 1.00	-59.469 < 0.001	-4.482 ~ -4.194. ↓	-2.25	-57.824 < 0.001	-4.362 ~ -4.074. ↓
AA	15.09 ± 4.68	18–1/3age*(= 11.8)	6.179 < 0.001	2.515 ~ 4.313 ↑	-	-	-

*NLP* Near lateral phoria, *DLP* Distance lateral phoria, *AC/A* Accommodative Convergence/ accommodation, *NFD_H* Near Horizontal Fixation Disparity, *DBI* Distance base-in, *DBO* Distance base-out, *NBI* Near base-in, *NBO* Near base-out,

Although the mean binocular visual function assessment values for young people in Taiwan were lower than the traditional standard values, in fact, the proportion of participants expressing distress or disturbance was low (21.7%). Furthermore, it should be noted that the standard deviations were quite large, reflecting heterogeneity among participants; therefore, variations in visual habits or visual demands might be an interesting topic for further research. Additionally, the results can be interpreted as reflecting a lower demand for binocular vision in visual work due to improved clarity of printed materials, the evolution of accessibility interfaces for 3C products, or the development of diversified sports and games. Future research should determine whether these differences resulted from binocular visual impairment or lower demand for binocular vision, with emphasis on applicability in clinical settings.

### Cutoff criteria of binocular visual functions for different visual tasks

In this study, the four dimensions of the visual behavior performance, namely, (1) near work, (2) perception, (3) comfort, and (4) physical balance, were scored according to quartiles. The first (lowest) quartile represented the 25th percentile of the data. This analysis was conducted to identify the presence or absence of symptoms. According to the different dimensions, participants were divided into the asymptomatic and symptomatic groups. Previous studies have confirmed that with this classification method, through t-test and logistic regression analysis, the questionnaire scores of each visual task can effectively predict the problems associated with binocular vision, and they can also have a significant amount of explained variance [49]. Next, the results of the binocular visual function testing were analyzed by using ROC curve analysis to identify the binocular vision appraisal items and standards for different visual tasks.

In terms of the mean total CSMU-VBP scores, t-test analysis on binocular visual functions revealed DBO recovery and BAF to be significantly different between the asymptomatic and symptomatic groups (Table 4). In the ROC curve analysis of the total scale, the base-out recovery (AUC = 0.619, *p* = 0.018) and binocular accommodative facilities (AUC = 0.588, *p* = 0. 605) could significantly identify visual behavior performance of the participants. The cutoff value of base-out recovery was 5.5 PD (sensitivity = 0.605, specificity = 0.606; Morgan and Scheiman and OEP standard both 10 PD). The cutoff value of binocular accommodative facility was 10.25 cpm (sensitivity = 0.588, specificity = 0.605; Scheiman standard = 10 cpm). The t-test and ROC analysis results were consistent with one another.

**Table 4:** t-test on binocular visual function between asymptomatic and symptomatic groups_CSMU-VBP_total score (*p < 0.05)

Examination	asymptomatic Mean ± SD	symptomatic Mean ± SD	*t* *(p*-value)	Examination	asymptomatic Mean ± SD	symptomatic Mean ± SD	*t* *p*-value
DLP	-1.700 ± 3.014	-2.159 ± 4.100	1.001 (0.318)	NBI_break	18.398 ± 6.146	18.093 ± 7.708	0.290 (0.772)
NLP	5.82 exophoria ± 6.52	6.03 exophoria ± 7.97	0.193 (0.847)	NBI_recovery	11.220 ± 5.309	10.767 ± 6.539	0.500 (0.617)
NFD_H	0.79 exophoria ± 2.08	1.06 exophoria ± 2.47	0.743 (0.458)	NBO_blur	4.250 ± 7.129	4.093 ± 7.745	0.132 (0.895)
Gradient AC/A(plus)	2.667 ± 4.382	1.953 ± 7.511	0.879 (0.380)	NBO_break	16.027 ± 6.913	14.128 ± 6.271	1.618 (0.107)
Gradient AC/A(minus)	2.454 ± 4.507	3.384 ± 5.548	-1.211 (0.227)	NBO_recovery	7.301 ± 5.529	6.231 ± 5.044	1.139 (0.255)
DBI_break	11.204 ± 3.655	11.209 ± 3.755	0.480 (0.632)	NPC_break	6.150 ± 2.413	6.233 ± 5.044	-0.187 (0.852)
DBI_recovery	4.319 ± 2.483	4.661 ± 2.816	-1.150 (0.251)	MAF	9.568 ± 5.111	8.744 ± 4.467	0.997 (0.320)
DBO_blur	6.70 ± 7.130	5.516 ± 6.991	1.062 (0.289)	BAF	10.131 ± 4.441	8.698 ± 4.086	1.984* (0.048)
DBO_break	18.401 ± 7.669	16.483 ± 8.388	1.167 (0.244)	NRA	1.721 ± 0.618	1.640 ± 0.565	0.809 (0.419)
DBO_recovery	6.374 ± 4.111	5.509 ± 3.675	2.334* (0.020)	PRA	-1.985 ± 1.300	-1.907 ± 1.272	-0.366 (0.715)
NBI_blur	6.750 ± 7.069	7.163 ± 8.306	-0.346 (0.729)	AA	14.977 ± 4.528	15.766 ± 5.553	-1.024 (0.307)

#### Near work

In the ROC curve analysis of the near-work dimension, the cutoff point for DBO blur (AUC = 0.598, *p* = 0.028) was 7 degrees (sensitivity = 0.74, specificity = 0.471; Morgan and Scheiman standard = 9). The cutoff point for DBO recovery (AUC = 0.591, *p* = 0.052) was 5.5 degrees (sensitivity = 0.543, specificity = 0.601; Morgan and Scheiman standard = 10 PD). Poor ability to revert to fusion was more likely to cause complaints about near work. This was similar to the criterion of pure exophoria (5 degrees) in the Scheiman binocular visual dysfunction diagnostic criteria. Therefore, these two functions should have considerable discriminative power when used to predict the conscious performance of the near-work dimension (Table 5).

**Table 5:** The standard binocular visual function values of near work and perceptual dimension (* significant)

CSMU-VBP questionnaire	Near work					Perceptual dimension				
	AUC	*p*-value	Sensitivity	Specificity	Cutoff point	AUC	*p*-value	Sensitivity	Specificity	Cutoff point
DLP	.534	.441	0.471	0.646	< − 2.25	.464	.463	0.146	0.94	< − 6.75
NLP	.524	.587	0.294	0.805	< − 10.5	.478	.646	0.268	0.798	< − 10.5
NFD_H	.473	.553	0.12	0.902	< − 2.5	.500	.995	0.098	0.966	< − 5.0
Gradient AC/A(plus)	.519	.672	0.412	0.66	> 3.75	.548	.324	0.366	0.771	> 4.75
Gradient AC/A(minus)	.540	.366	0.451	0.663	> 3.75	.517	.726	0.463	0.66	> 3.75
DBI_break	.505	.916	0.3	0.763	< 8.5	.548	.330	0.325	0.764	< 8.5
DBI_recovery	.468	.479	0.12	0.969	< 1.5	.504	.932	0.35	0.707	< 2.5
DBO_blur	**.598**	.028*	**0.74**	**0.471**	** < 7**	.504	.931	0.7	0.348	< 9.0
DBO_break	.565	.154	0.583	0.586	< 15	.513	.796	0.579	0.579	< 15.0
DBO_recovery	**.591**	.050*	**0.543**	**0.601**	** < 5.5**	.569	.180	0.917	0.23	< 8.5
NBI_blur	.472	.528	0.765	0.234	< 13	.459	.394	0.756	0.233	< 13.0
NBI_break	.549	.270	0.471	0.625	< 16.5	.539	.426	0.61	0.5	< 18.5
NBI_recovery	.542	.340	0.353	0.77	< 7.5	.546	.342	0.585	0.534	< 10.5
NBO_blur	.511	.812	0.824	0.258	< 9	.479	.667	0.829	0.184	< 11.0
NBO_break	.555	.228	0.277	0.833	< 9.5	.560	.234	0.595	0.523	< 14.5
NBO_recovery	.532	.484	0.936	0.147	< 13	.496	.941	0.162	0.916	< 1.5
NPC_break	.466	.437	0.059	0.976	> 12.5	.576	.119	0.78	0.336	< 6.25
MAF	.532	.467	0.804	0.275	< 12.5	**.583**	.088*	**0.756**	**0.419**	** < 10.5**
BAF	.550	.258	0.941	0.217	< 13.5	**.621**	.012*	**0.707**	**0.481**	** < 10.25**
NRA	.535	.428	0.529	0.584	< 1.63	.497	.954	0.805	0.273	< 2.13
PRA	.529	.515	0.373	0.716	> − 1.13	.493	.893	0.854	0.184	> − 3.13
AA	.536	.422	0.824	0.29	> 12.43	.520	.679	0.805	0.283	< 12.43

#### Perceptual

In the ROC curve analysis of the perceptual dimension (Table 5 right), the cutoff point for MAF (AUC = 0.583, *p* = 0.088) was 10.5 cpm (sensitivity = 0.756, specificity = 0.419; Scheiman criterion = 11 cpm). The cutoff for BAF (AUC = 0.621, *p* = 0.012) was 10.25 cpm (sensitivity = 0.70, specificity = 0.481; Scheiman standard = 10 cpm). Individuals with good monocular and binocular accommodation ability can switch between binocular distance and near vision with high levels of smoothness and comfort, and their vision is more stable and not too strained. Such individuals can focus more on cognition and learning [54, 55]. In contrast, individuals with poor binocular accommodation facilities find it very difficult and experience unstable vision when looking at objects of varying distances, with excessive mental effort needed to adjust vision; thus, they find it difficult to focus their working attention; moreover, they experience much difficulty not only in three-dimensional perception [56], but also in visual recognition and comprehension [57]. The cutoff point (10.25 cpm) analyzed in this study is close to the traditional standard value (10 cpm); therefore, MAF and BAF functions can be used to predict the perception dimension, and conscious performance should have facilitated considerable discrimination.

#### Comfort

In the ROC curve analysis of comfort (Table 6), the cutoff point for DLP (AUC = 0.567, *p* = 0.050) was 2.25 PD of exophoria (sensitivity = 0.459, specificity = 0.673; Morgan and Scheiman criterion = 1 PD of exophoria; OEP standard = 0.5 PD of exophoria). The cutoff point for DBI break (AUC = 0.574, *p* = 0.031) was 11.5 PD (sensitivity = 0.593, specificity = 0.563; Morgan and Scheiman standard = 7 PD; OEP standard = 9 PD). The cutoff for NBI blur (AUC = 0.561, *p* = 0.039) was 15 PD (sensitivity degree = 0.229, specificity = 0.884; Morgan and Scheiman standard = 13 PD; OEP standard = 14 PD). For NBI break (AUC = 0.585, *p* = 0.014), the cutoff was 17.5 PD (sensitivity = 0.697, specificity = 0.449; Morgan and Scheiman standard = 21 PD; OEP standard = 22 PD). The cutoff value for NBI recovery (AUC = 0.581, *p* = 0.019) was 13 PD (sensitivity = 0.422, specificity = 0.717; Morgan and Scheiman standard = 13 PD; OEP criteria = 18 PD). For NPC, the cutoff point 5.75 cm (AUC = 0.570, *p* = 0.042, sensitivity = 0.679, specificity = 0.452; Scheiman standard = 5 cm). The six above-mentioned binocular vision criteria can significantly identify the comfort dimension with regard to an easy starting point and appropriate convergence and divergence abilities, both in terms of far and near vision. This can allow the eyes to easily relax and retract [58–60]. Therefore, these six binocular visual function items can be used to predict the conscious performance of the comfort dimension with considerable discriminating power.

**Table 6:** The standard binocular visual function values of comfort and balance dimensions (* significant)

CSMU-VBP questionnaire	Comfort					Balance				
	AUC	*p*-value	Sensitivity	Specificity	Cutoff point	AUC	*p*-value	Sensitivity	Specificity	Cutoff point
DLP	**.567**	.050*	**0.459**	**0.673**	** < ** − **2.25**	.447	.245	0.064	0.977	< − 8.5
NLP	.548	.163	0.468	0.643	< − 6.25	.434	.149	0.128	0.927	< − 16.5
NFD_H	.581	.581	0.343	0.69	< − 1.5	**.629**	.005*	**0.809**	**0.368**	** > ** − **0.5**
Gradient AC/A(plus)	.503	.934	0.672	0.376	< 1.75	.528	.544	0.34	0.769	> 4.75
Gradient AC/A(minus)	.536	.296	0.321	0.766	> 4.25	**.590**	.051*	**0.652**	**0.558**	** > 2.25**
DBI_break	**.574**	.031*	**0.593**	**0.563**	** > 11.5**	.544	.332	0.362	0.769	< 9.5
DBI_recovery	.471	.402	0.065	0.965	< 1.5	.491	.853	0.348	0.708	< 2.5
DBO_blur	.494	.858	0.75	0.291	< 11.0	.571	.123	0.723	0.465	< 7.0
DBO_break	.486	.682	0.107	0.907	< 7.5	.530	.529	0.512	0.571	< 15.0
DBO_recovery	.529	.414	0.263	0.795	< 2.5	.560	.214	0.548	0.599	< 5.5
NBI_blur	**.561**	.077*	**0.229**	**0.884**	** > 15.0**	.447	.248	0.745	0.231	< 13.0
NBI_break	**.585**	.014*	**0.697**	**0.449**	** > 17.5**	.548	.297	0.596	0.5	< 18.5
NBI_recovery	**.581**	.019*	**0.422**	**0.717**	** > 13.0**	.523	.612	0.234	0.896	< 4.5
NBO_blur	.504	.909	0.771	0.253	< 9.0	.506	.889	0.979	0.081	< 17.0
NBO_break	.528	.428	0.272	0.791	< 11.0	.470	.532	0.233	0.77	< 11.0
NBO_recovery	.510	.767	0.806	0.241	< 11.0	.426	.120	0.116	0.91	< 1.5
NPC_break	**.570**	.042*	**0.679**	**0.452**	** < 5.75**	**.584**	.066*	**0.596**	**0.591**	** < 4.75**
MAF	.529	.401	0.376	0.695	< 7.5	.502	.970	0.652	0.404	< 10.5
BAF	.522	.518	0.333	0.756	< 7.5	.523	.614	0.957	0.054	< 16.5
NRA	.489	.749	0.33	0.719	< 1.375	.485	.741	0.34	0.709	< 1.375
PRA	.539	.260	0.56	0.523	< − 1.63	.504	.923	0.872	0.188	> − 3.125
AA	.548	.169	0.407	0.687	> 16.56	.534	.464	0.809	0.286	> 12.43

#### Balance

In the ROC curve analysis of the balance dimension (Table 6), the cutoff point for NFD (AUC = 0.629, *p* = 0.005) was 0.5 PD of exotropic shift (sensitivity = 0.809, specificity = 0.368; the standard value of an optometry practical textbook is ortho). The cutoff point for the negative gradient AC/A was 2.25△/D (AUC = 0.590, *p* = 0.050; sensitivity = 0.652, specificity = 0.558; Morgan and Scheiman criterion = 4Δ/D; OEP criterion = 4Δ/D). For NPC (AUC = 0.584, *p* = 0.033), the cutoff was 4.75 cm (sensitivity = 0.596, specificity = 0.591; Scheiman standard = 5 cm). NFD represents the accuracy and stability of near gaze. The standard (0.5 PD exotropic shift) analyzed in this study was similar to the standard value (ortho) specified in an optometry clinical practice textbook. The results indicated that the deviation of NFD was higher than 0.5 exotropic PD when the direction of the esotropic NDF increased, and balance-related complaints increased simultaneously [61]. Gradient AC/A (negative) indicates that convergence will be generated under the stimulation of 1D accommodation. The cutoff node (2.25△/D) analyzed in the study was 1.75△/D less than the traditional standard value (4△/D), which means that as long as the gradient AC/A (negative) ratio of the subject exceeds 2.25△/D, there will be significant complaints about balance-related symptoms [62]; the converging near point represents the maximum amplitude that can be converged [63], and the cutoff point (4.75 cm) determined by this study was close to the Scheiman standard value of 5 cm, which means that the convergence point is related to postural balance [62]. Postural balance ability correlated with NFD, negative gradient AC/A, and NPC.

## Discussion

According to the analysis of the results of the binocular visual function examination in this study, the proportion of accommodation excess and convergence excess among young adults in this study were the highest, which is similar to the results reported elsewhere [64–67]. However, the findings of this study are very different from those of most previous studies with regard to accommodation and convergence insufficiency [9, 68, 69]. Previous research has suggested that long-term use of 3C products at close visual range [70–73] results in myopia and an inability to relax accommodation and convergence; this might reasonably to explain the diagnoses of accommodation and convergence excesses in Taiwanese youths [67, 74]. However, the classical diagnostic criteria for the clinical assessment and classification of abnormal binocular visual function are nearly 80 years old, and many reports have confirmed race- and age-specific observations [15, 16, 18, 20, 75–78]; therefore, the diagnostic criteria for binocular vision dysfunction should be re-evaluated [16–19].

**Fig. 2:**
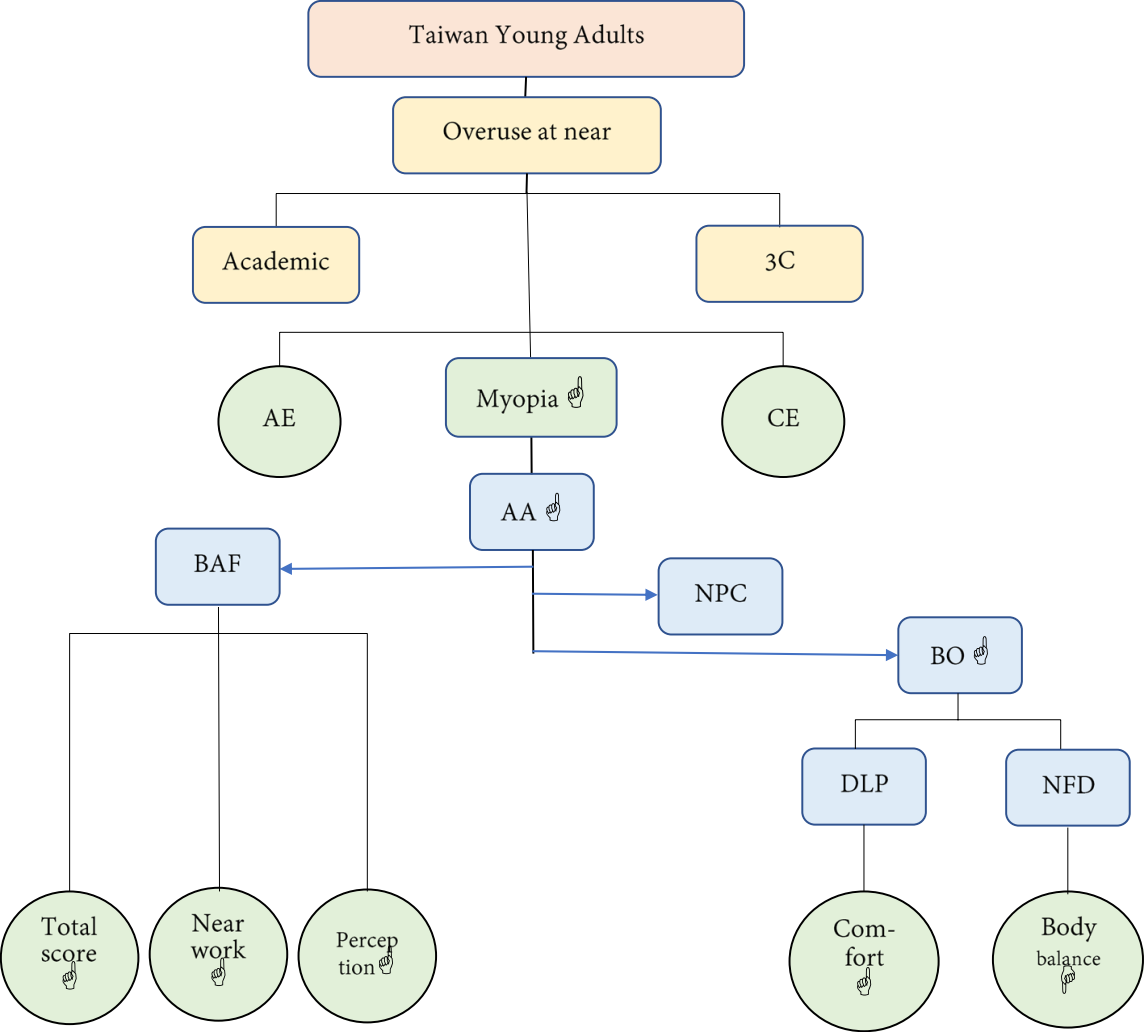
Binocular visual relationship diagram related to various visual tasks

The binocular visual functional outcome values of young Taiwanese diverged from the standard values that are often referred to in clinical practice, and the actual values were mostly lower than the standard values. Except for established ethnic differences and age differences [15, 16, 18, 20, 75–78], most of the differences from standard values did not take refractive errors, visual demands, and technological developments into consideration [79–81]. Although it might not confer reading difficulties [82–84] when the accommodation range and the DBI break are both relatively high, overuse of near vision as well as excessive use of accommodation and cohesion, resulting in functional fatigue or rigidity, may lead to poor overall binocular visual performance (Fig. 2). Regardless of the study findings, it can be confirmed that the development of new Taiwanese binocular vision standard values should constitute a direction for future research.

In this study, ROC curve analysis was used to identify standard binocular vision criterion values for different visual behavior performance dimensions. When patients have visual disturbances, the visual behavior scale questionnaire can be used to distinguish between different categories of patients. After identification of the type of visual disturbance in each dimension, binocular vision inspection items can be used to discriminate each dimension to determine whether measured and calculated values meet diagnostic criteria (cutoff points). Next, the values thus obtained can be used to adjust prescriptions or vision training [85–88] to help patients resolve or manage their visual problems. For example, the patient fills in the CSMU-Visual Behavioral Performance questionnaire, and the score in the perception dimension is 7 points (≥ 5 is symptomatic), which reflects perceptual visual disturbance. The results of monocular and binocular accommodation evaluations are 8 and 7 cpm, respectively (lower than the cutoffs for MAF [< 10.5 cpm] and BAF [< 10.25 cpm]). The examiner can preliminarily determine that the patient has abnormal function in monocular and binocular accommodation. By improving binocular visual function, the perceptual ability can also be improved simultaneously. Figure 2 is a simplified representative diagram, which indicates that different visual tasks correlated with different binocular visual functions [89].

Comparing Tables 3 and 4, it can be found that although DBO recovery and BAF were significantly different between the asymptomatic and symptomatic groups, the DBO recovery values of the two groups were both lower than the standard value. In contrast, the BAF performance of all participants was not significantly different from the standard value, but there was a significant difference between the two groups, which indicates that BAF is the most important indicator for clinical diagnosis.

In summary (Fig. 2), a high frequency of near work among Taiwanese young adults has increased the values for the following variables: accommodation range (AA↑), near point of convergence (NPC↑), distance-near convergence ability (BO↑), and binocular accommodation facility (BAF↑). However, excessive near work and related habits leads to excessive accommodation and convergence, which leaves individuals exhausted and fatigued, unable to relax, resulting in poor binocular visual function. Therefore, such people will have more visual disturbance–related complaints than those who do not meet the standard values for accommodation and convergence. If the starting point of eye positioning is close to ortho (DLPΦ) at distance, the endpoints of near fixation disparity close to exotropia (NFD↓), good binocular accommodation facility (BAF↑), and visual behavior disturbance symptoms will be naturally better.

## Conclusions

The average binocular visual function of young people in Taiwan is worse than that which would be expected according to traditional reference values. When using the traditional binocular visual function classification criteria, nearly 80% of the patients diagnosed with binocular vision abnormality have no obvious symptoms. Visual habits and demands vary with technological and scientific advancements over time and with racial differences. Moreover, future research should focus on related topics regarding the development of human interface technology products, as well as whether these findings reflect binocular vision abilities or demands. Binocular visual functions, especially binocular accommodation sensitivity, should be part of routine optometric examinations to ascertain patients' visual behavior performance.

Finally, this study analyzed the binocular visual function of Taiwanese youths based on questionnaires and binocular vision examinations; however, there are some limitations owing to the large difference in the proportion of male and female participants in this study, as well as the validity and application of the CSMU-VBP questionnaire in clinical practice.

## Supplementary Information

**Additional file 1.** CSMU - Visual Behavioral Performance

## Data Availability

All data generated or analyzed during this study are included in this published article and its supplementary information files. Correspondence and requests for materials should be addressed to C.-Y.C.
